# Risk Factors of Exercise Intolerance in Children and Adolescents After Total Cavopulmonary Connection

**DOI:** 10.3390/jcdd13050188

**Published:** 2026-04-29

**Authors:** Xuwen Xiao, Ge Fan, Haifan Wen, Xiaowei Li, Min Tong, Ruyin Guo, Yixin Liao, Ma Li, Yanqin Cui

**Affiliations:** 1 Medical Education Center of Jinan University, Jinan University, Guangzhou 510632, China; xiaoxuwen@gwcmc.org; 2 Department of Surgical Intensive Care Unit (SICU), Guangzhou Women and Children’s Medical Center, Guangzhou Medical University, Guangzhou 510623, China; fange@gwcmc.org (G.F.); wenhf8@hyhospital.com (H.W.); lixiaowei@gwcmc.org (X.L.); tongmin@gwcmc.org (M.T.); guoruying@gwcmc.org (R.G.); liaoyixin@gwcmc.org (Y.L.); 3 Department of Cardiac Intensive Care Unit (CICU), Guangzhou Women and Children’s Medical Center, Guangzhou Medical University, Guangzhou 510623, China

**Keywords:** TCPC, exercise intolerance, CPET, risk factors, pediatrics

## Abstract

(1) Background: Exercise intolerance is a nearly ubiquitous consequence among patients who have undergone the total cavopulmonary connection (TCPC) or modified Fontan procedure; however, the specific factors influencing this condition—especially the impact of surgical timing and perioperative management—remain inadequately understood. (2) Methods: From a retrospective cohort of 255 TCPC patients, 99 who had undergone cardiopulmonary exercise testing (CPET) were included. Patients were stratified into a normal exercise tolerance group (ET group, peak VO_2_ ≥ 80% predicted, *n* = 45) and an exercise intolerance group (EI group, peak VO_2_ < 80% predicted, *n* = 54). Univariate and multivariate logistic regression analyses were performed to identify risk factors for decreased exercise tolerance. (3) Results: Patients in EI group were older at TCPC surgery (4.67 vs. 3.60 years, *p* = 0.004) and CPET (10.11 vs. 8.65 years, *p* = 0.01). They had longer operation time (312.14 vs. 267.53 min, *p* = 0.01), bypass time (122.26 vs. 103.59 min, *p* = 0.045), and higher rate of prolonged ICU stay (>3 days, 68.9% vs. 31.1%, *p* = 0.01). ROC analysis determined optimal surgical age cut-off at 3.81 years (AUC = 0.692, *p* = 0.001). Univariate analysis identified age ≥ 3.8 years, ICU stay > 3 days, longer operation/bypass times, and postoperative atrioventricular valve regurgitation as significant risk factors. Multivariate analysis confirmed age at TCPC ≥3.8 years (OR = 2.54, 95% CI: 1.01–6.42, *p* = 0.049) and ICU stay >3 days (OR = 2.61, 95% CI: 1.07–6.39, *p* = 0.04) as independent predictors of exercise intolerance. (4) Conclusions: The delayed completion (beyond 3.8 years of age) of TCPC procedure and the occurrence of early postoperative complications are associated with reduced long-term exercise capacity.

## 1. Introduction

The total cavo pulmonary connection (TCPC), or modified Fontan procedure, represents the final stage of palliative surgery for children born with single ventricle physiology [1]. This surgical strategy, by creating a circulation in series, successfully separates the systemic and pulmonary circuits, thereby alleviating cyanosis and reducing the chronic volume overload on the single ventricle. As a result, both the medium- and long-term survival rates of these patients have improved significantly over the past decades. 

Despite its success, the Fontan circulation is inherently precarious, characterized by the absence of a subpulmonary ventricular pump. This passive flow system predisposes patients to a spectrum of long-term complications, including progressive ventricular dysfunction, protein-losing enteropathy, and liver disease [2,3,4]. A central manifestation of this compromised physiology is “exercise intolerance”, which is nearly universal in this population and is a powerful predictor of morbidity, hospitalizations, and mortality [5,6,7]. Cardiopulmonary exercise testing (CPET) has emerged as the gold standard for objectively quantifying functional capacity, with peak oxygen consumption (VO_2_) serving as a key prognostic marker [8,9,10].

While exercise limitation is common, its severity varies markedly among Fontan survivors. A subset of patients, often termed “Super-Fontan,” exhibits remarkably preserved exercise capacity, while others experience significant decline [11,12]. Identifying the factors that predispose patients to poor functional outcomes is therefore critically important [13,14]. Previous research has explored the roles of ventricular morphology, systolic dysfunction, and elevated pulmonary vascular resistance [6,14,15,16]. However, the impact of specific “surgical timing and perioperative course factors”—such as age at TCPC completion, operative duration, and postoperative recovery—on long-term exercise capacity remains less well characterized and is often conflicting, partly due to heterogeneous study populations and methodologies [17,18].

Therefore, the primary objective of this single-center, retrospective cohort study was to systematically investigate the demographic, anatomical, and perioperative determinants of exercise intolerance in children and adolescents with functional single ventricle after the TCPC procedures.

## 2. Materials and Methods

### 2.1. Ethical Approval and Patient Consent

The study was approved by the Institutional Review Board (Approval No. [GZWCMC-IRB-2024-372B00]). All data were retrieved from the electronic medical record system. Written and verbal informed consent had been obtained from all participants (or their legal guardians) for the clinically indicated cardiopulmonary exercise testing (CPET) procedures.

### 2.2. Patient Selection

**Figure 1:**
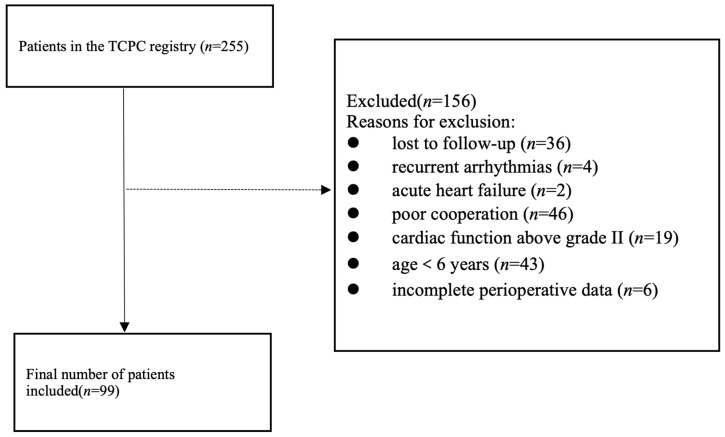
Study design and patient selection flow chart. This figure illustrates the stepwise exclusion process from the initial TCPC cohort (*n* = 255) to the final analytical cohort (*n* = 99).

A single-center, retrospective cohort study was conducted among 255 consecutive patients who underwent TCPC completion at this institution between 2010 and 2024. After excluding 36 patients lost to follow-up or deceased (Lost to follow-up was defined as no outpatient clinic visit for ≥2 years by the data cutoff date), 219 patients remained in regular follow-up in our specialized congenital heart disease clinic. The study cohort was subsequently refined through systematic exclusion criteria: patients with recurrent arrhythmias (*n* = 4) or acute heart failure (*n* = 2); those unable to undergo cardiopulmonary exercise testing (CPET) due to financial constraints or poor cooperation (*n* = 46); patients with cardiac function grade III or IV (*n* = 19); and children below 6 years of age (*n* = 43); and incomplete perioperative data (*n* = 6). The final analytical cohort comprised 99 patients who had successfully completed at least one CPET examination (defined as achieving a respiratory exchange ratio (RER) > 1.00 [19]), forming the TCPC group for subsequent analysis (Figure 1). Data were systematically extracted from electronic medical records and surgical databases. The collected variables were categorized as follows:

### 2.3. Demographic and Preoperative Data

Age at TCPC surgery, gender, preoperative height, weight, body mass index (BMI), and body surface area (BSA). Preoperative clinical diagnoses were recorded, including ventricular morphology (left ventricular dominance, right ventricular dominance, biventricular, indeterminate), associated conditions (pulmonary hypertension, pulmonary stenosis/atresia, visceral heterotaxy syndrome). Preoperative peripheral oxygen saturation was also recorded.

### 2.4. Intraoperative Data

Key surgical parameters included the performance of an intraoperative fenestration, total operation duration (from skin incision to closure, in minutes), and total cardiopulmonary bypass time (in minutes).

### 2.5. Postoperative Data

Postoperative outcomes included: case numbers of mechanical ventilation support time ≥ 48 h, intensive care unit (ICU) stay > 3 days, chest drainage tube duration >14 days or the occurrence of chylothorax, total postoperative hospital stay >14 days, the need for postoperative reoperation, unplanned postoperative hospitalizations, the presence of postoperative electrocardiogram (ECG) abnormalities, the development of postoperative complications (a composite variable including poor wound healing, arrhythmias, thrombosis, recurrent hypoalbuminemia, decreased ventricular function, hemoptysis, or death), the presence of moderate or severe atrioventricular valve regurgitation at the most recent follow-up echocardiogram prior to CPET and the most recent pre-CPET N-terminal pro-brain natriuretic peptide (NT-proBNP) level.

### 2.6. Exercise Tolerance Assessment

Cardiopulmonary exercise testing (CPET) was performed as part of routine clinical follow-up. All tests were conducted using a standardized, symptom-limited, progressive ramp protocol on a motorized treadmill (GE Healthcare, Little Chalfont, UK). Bruce protocol or modified Bruce protocol, as suggested by the American College of Sports Medicine (ACSM), depending on the child’s age and functional status. During the test, patients were encouraged to exercise to volitional exhaustion. Continuous electrocardiographic monitoring was performed, and blood pressure was measured at regular intervals. Respiratory gas exchange analysiswas conducted breath-by-breath using a metabolic cart (MasterScreen CPX, Vyaire Medical, Hoechberg, Germany) to measure oxygen uptake (VO_2_), carbon dioxide output (VCO_2_), and minute ventilation. 

### 2.7. Group Stratification

Peak oxygen consumption (VO_2_) is the most widely accepted gold standard for assessing exercise capacity in Fontan patients. Therefore, we used percent-predicted peak VO_2_ as the primary criterion for group stratification. In this study, patients were stratified into two groups based on the results of their most recent CPET: Exercise Tolerance group (ET group): *n* = 45, Exercise intolerance Group (EI group): *n* = 54. The ET group was defined as achieving normal exercise and work capacity (peak VO_2_ ≥ 80% predicted) and the EI group consisted of Fontan subjects who had reduced exercise or work capacity (peak VO_2_ < 80% predicted) [20].

### 2.8. Follow-Up Time

Follow-up time was defined as the interval between the TCPC surgery and the latest CPET. The age at which the latest CPET was performed was also recorded.

### 2.9. Statistical Analysis

Continuous variables were tested for normality using the Shapiro–Wilk test. Normally distributed data were presented as mean ± standard deviation (SD) and compared using the independent samples *t*-test. Non-normally distributed data were presented as median with interquartile range (P25, P75) and compared using the Mann–Whitney U test. Categorical variables were expressed as frequencies and percentages and compared using the Chi-square test or Fisher’s exact test, as appropriate. Univariate logistic regression analysis was performed to identify factors associated with decreased exercise tolerance. Variables with a *p*-value < 0.10 in the univariate analysis were subsequently entered into a multivariate logistic regression model using a backward stepwise selection method to identify independent risk factors. The results were expressed as odds ratios (OR) with 95% confidence intervals (CI). A two-tailed *p*-value < 0.05 was considered statistically significant for all final analyses. All statistical analyses were performed using SPSS Statistics version 20 (IBM Corp., Armonk, NY, USA).

## 3. Results

### 3.1. Patient Characteristics and Clinical Profile

A total of 99 patients after TCPC procedures who underwent CPET were included in the final analysis. Among the group, the median surgical age was 3.96 (IQR: 3.09–5.45) years, with a median postoperative follow-up duration of 5.3 (IQR: 3.39–6.84) years. The cohort comprised approximately a 3:2 male-to-female ratio. All included patients were classified as New York Heart Association (NYHA) functional class I or II. There was one mortality (*n* = 1, 1.0%) recorded in the cohort.

### 3.2. Comparison of Demographic Characteristics

Based on the CPET results, the cohort was divided into an ET group (*n* = 45) and an EI group (*n* = 54). The comparison of demographic characteristics between the two groups was presented in Table 1.

Patients in the EI group were significantly older at the time of TCPC surgery [4.67 (3.36, 6.68) vs. 3.60 (2.97, 4.59) years, *p* = 0.004] and at the time of CPET [10.11 (7.68, 13.81) vs. 8.65 (7.01, 13.81) years, *p* = 0.01]. They also had significantly greater preoperative height [100 (93.75, 111) vs. 95 (90, 100) cm, *p* = 0.01] and weight [14.55 (13.28, 18.9) vs. 13.5 (12, 15.15) kg, *p* = 0.01], but a lower preoperative Body Surface Area (BSA) [0.58 (0.54, 0.65) vs. 0.63 (0.6, 0.77) m^2^, *p* = 0.02]. There were no significant differences in preoperative BMI, BMI/BSA before CPET, follow-up time, ventricular dominance, visceral status, or gender distribution between the two groups. In the ET group, 36 patients (80.0%) were NYHA class I and 9 (20.0%) were class II. In the EI group, 36 patients (66.7%) were NYHA class I and 18 (33.3%) were class II. The proportion of NYHA class I patients was higher in the ET group.

### 3.3. Comparison of Perioperative Conditions

The perioperative characteristics are detailed in Table 2. No significant differences were found between the two groups in terms of preoperative oxygen saturation, latest NT-proBNP level, prevalence of specific anatomical diagnoses (pulmonary hypertension, tricuspid atresia, visceral heterotaxy syndrome), or the rate of intraoperative fenestration. The EI group had a substantially longer operation duration (312.14 ± 89.79 vs. 267.53 ± 93.03 min, *p* = 0.01) and cardiopulmonary bypass time (122.26 ± 58.49 vs. 103.59 ± 48.46 min, *p* = 0.045). Furthermore, a prolonged ICU stay of >3 days was significantly more frequent in the EI group (68.9% vs. 31.1%, *p* = 0.01).

Postoperatively, the prevalence of moderate or severe atrioventricular valve regurgitation was significantly higher in the EI group (33.3% vs. 14.3%, *p* = 0.04). Although not reaching statistical significance, there were trends towards a higher incidence of postoperative ECG abnormalities (62.3% vs. 37.7%, *p* = 0.06), any postoperative complications (65.9% vs. 34.1%, *p* = 0.06), and a postoperative hospital stay >14 days (55.6% vs. 44.4%, *p* = 0.06) in the EI group.

### 3.4. Optimal Cut-Off Values for Age at Surgery as Predictors of Decreased Exercise Tolerance

**Figure 2:**
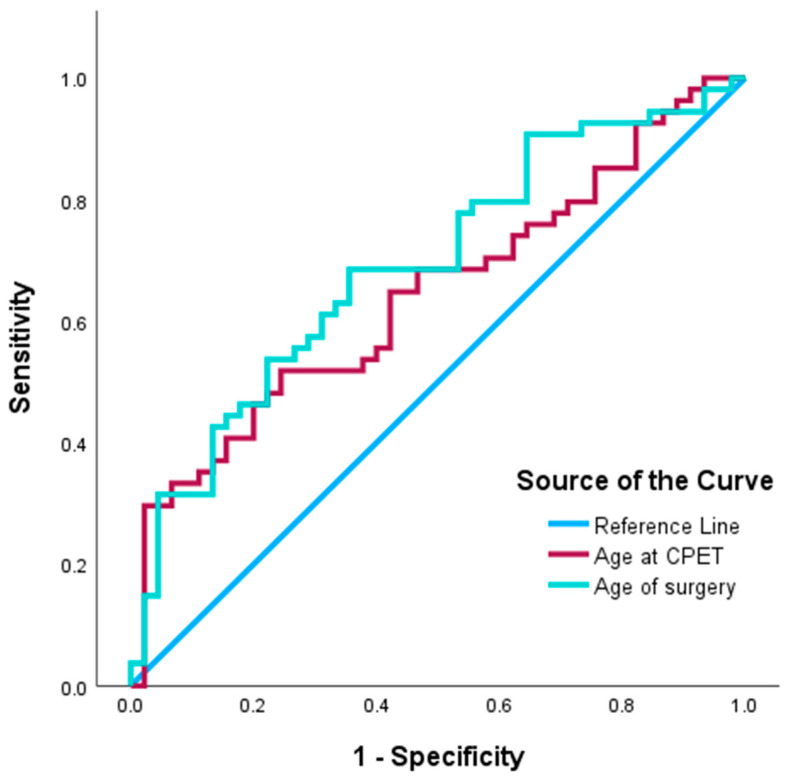
ROC Curve Analysis of Surgical Age and Examination Age.

Receiver operating characteristic (ROC) curve analysis was performed to determine the optimal cut-off points for continuous variables such as age at surgery and age at CPET. The results are shown in Figure 2 and Table 3 below. The area under the ROC curve (AUC) for age at surgery in predicting decreased exercise tolerance after TCPC was 0.692 (*p* = 0.001), with an optimal cut-off value of 3.81 years, a sensitivity of 0.69, and a specificity of 0.64. For age at CPET, the AUC was 0.644 (*p* = 0.014), with an optimal cut-off value of 9.93 years, a sensitivity of 0.52, and a specificity of 0.76.

### 3.5. Univariate and Multivariate Logistic Regression Analysis for Decreased Exercise Tolerance

The results of the univariate and multivariate logistic regression analyses are shown in Table 4.

Univariate analysis identified several significant risk factors for decreased exercise tolerance: age at surgery ≥ 3.8 years (OR = 3.95, 95% CI: 1.71–9.72, *p* = 0.001), age at CPET ≥ 10 years (OR = 3.33, 95% CI: 1.40–7.90, *p* = 0.006), operation duration ≥ 237.5 min (OR = 5.35, 95% CI: 2.20–13.00, *p* < 0.001), cardiopulmonary bypass time ≥ 77.5 min (OR = 3.33, 95% CI: 1.31–8.46, *p* = 0.01), ICU stay > 3 days (OR = 2.98, 95% CI: 1.30–6.85, *p* = 0.01), and postoperative moderate or severe AV valve regurgitation (OR = 4.30, 95% CI: 0.88–21.05, *p* = 0.048).

In the multivariate analysis, which adjusted for confounding factors, two variables remained independent predictors of decreased exercise tolerance: age at surgery ≥ 3.8 years (OR = 2.54, 95% CI: 1.01–6.42, *p* = 0.049) and ICU stay > 3 days (OR = 2.61, 95% CI: 1.07–6.39, *p* = 0.04). The effects of operation duration, cardiopulmonary bypass time, and AV valve regurgitation were attenuated and no longer statistically significant in the multivariate model.

## 4. Discussion

By stratifying 99 patients into “Super-Fontan” and decreased exercise tolerance groups based on objective cardiopulmonary exercise testing (CPET), we identified a profile of demographic and perioperative factors that portend a poorer functional outcome. Our analysis of 99 TCPC patients reveals that the timing of the operation and the complexity of the postoperative course, rather than anatomical factors alone, are key independent drivers of functional decline. Specifically, we identified age at TCPC ≥ 3.8 years and ICU stay > 3 days as robust predictors, suggesting that delays in establishing the Fontan circulation and early postoperative morbidity have lasting consequences on cardiopulmonary reserve. The following discussion interprets these key findings in the context of existing literature and the specific results of this study.

### 4.1. The Critical Impact of Age at Surgery and at Assessment

A central finding of this study is the strong association between older age and worse exercise performance. This finding aligns robustly with the established physiological rationale for staged palliation in single-ventricle physiology. The pre-Fontan stages (the bidirectional Glenn procedure) are characterized by a state of chronic cyanosis and volume overload on the single ventricle. A delay in completing the Fontan circulation prolongs this suboptimal physiological state, potentially leading to irreversible changes in ventricular geometry, myocardial fibrosis, and impaired development of the pulmonary vascular bed [21,22]. An earlier TCPC, performed before these detrimental adaptations become entrenched, may allow for more favorable ventricular remodeling and better preservation of systolic and diastolic function [17]. Consequently, these patients enter adolescence with a superior cardiopulmonary reserve, which is directly reflected in their CPET parameters, particularly peak VO_2_%. The correlation between older age at CPET and decreased tolerance further suggests that the Fontan circulation’s inherent limitations—such as the lack of a subpulmonary ventricular pump and its dependence on low pulmonary vascular resistance—become more pronounced as the body’s metabolic demands increase with growth and age [23].

### 4.2. External Validity of the ≥3.8-Year Threshold

Compared with multi-center registries, our single-center finding that TCPC completion beyond 3.8 years is associated with a 2.5-fold higher risk of late exercise intolerance aligns closely with the recently published National Institutes of Health/National Heart, Lung and Blood Institute Pediatric Heart Network(PHN) Fontan cohorts and Australia-New Zealand Fontan (ANSWER) cohorts. In the PHN Fontan Cross-Sectional Study of 405 Fontan survivors, in patients who reached VAT, each year increase in age at Fontan completion was associated with a decline of 1.5 (95% CI −2.5 to −0.5) points in percent-predicted VO_2_ maximum [24]. Similarly, the ANSWER longitudinal series showed that every 1-year delay beyond 3.5 years reduced final adult peak VO_2_ by 1.2% predicted (95% CI 0.4 to 2.0) [12]. The present ROC-derived cut-off of 3.8 years therefore falls squarely within the “risk inflection window” identified by these much larger, multi-center databases, providing external corroboration for our local observations despite differences in ethnicity, surgical strategy and CPET protocols. This congruence also suggests that the 3.8-year threshold may be generalizable and could be used as a pragmatic quality indicator when planning staged palliation in other populations. 

### 4.3. Perioperative Course as a Harbinger of Long-Term Functional Decline

Our analysis revealed significant perioperative disparities between the two groups. The decreased exercise tolerance group experienced substantially longer operation durations and cardiopulmonary bypass (CPB) times. While these factors were significant in univariate analysis, they were attenuated in the multivariate model. In contrast, a prolonged ICU stay of more than three days emerged as a powerful and independent risk factor (OR = 2.61). The longer operation and CPB times are indicative of greater surgical complexity, potentially due to intricate anatomy, previous adhesions, or the need for concomitant procedures like atrioventricular valve repair. Prolonged CPB is a well-documented driver of a systemic inflammatory response, which can cause capillary leak, myocardial edema, and end-organ dysfunction, potentially impairing both immediate recovery and long-term myocardial performance [25]. However, the fact that ICU stay remained significant after adjustment suggests that it serves as a more comprehensive, composite marker of a “difficult postoperative course.” An extended ICU stay typically results from complications such as low cardiac output syndrome, significant pleural effusions or chylothorax, arrhythmias, or infections—all of which can directly injure the myocardium and pulmonary vasculature. Furthermore, the period of enforced sedation, immobilization, and mechanical ventilation during a lengthy ICU admission leads to rapid skeletal muscle deconditioning and diaphragmatic weakness [25,26]. This acquired myopathy can severely limit exercise capacity independently of cardiac function, creating a legacy of physical impairment that persists long after hospital discharge.

### 4.4. The Role of Postoperative Cardiac Morphology and Function

The univariate analysis identified postoperative moderate or severe atrioventricular valve regurgitation (AVVR) as a significant risk factor (OR = 4.30). This is mechanistically sound, as AVVR in the Fontan circulation creates a volume-loading burden on the single ventricle. This regurgitant loop leads to elevated atrial pressures, pulmonary venous congestion, and reduced effective forward flow, severely constraining the ability to increase cardiac output during exercise [27]. The fact that it did not retain independent significance in the multivariate model is intriguing. This may be due to several factors: the sample size may have been insufficient to detect its independent effect amidst other strong predictors; it may share collinearity with other variables like older age at surgery (as a diseased ventricle may be more prone to annular dilation and regurgitation); or its impact may be partially mediated through other pathways captured by variables like prolonged ICU stay (e.g., a patient with significant AVVR may have a more complicated postoperative course).

Similarly, the strong trends observed towards higher rates of postoperative ECG abnormalities and overall complications in the decreased exercise tolerance group (*p* = 0.06) underscore that a complicated clinical trajectory, from the operating room through the entire hospitalization, is a hallmark of patients destined for poorer long-term functional outcomes.

### 4.5. Limitations

Several limitations of this study must be acknowledged. First, its retrospective and single-center design inherently carries risks of selection bias and unmeasured confounding. Factors known to influence exercise capacity, such as habitual physical activity levels, nutritional status, socioeconomic background, and psychosocial factors, were not accounted for. Second, while the sample size is substantial for a single-center Fontan cohort, it may still limit the statistical power to identify all but the strongest independent predictors, potentially leading to Type II errors. Third, the CPET was performed as part of routine clinical follow-up, leading to variability in the timing of the assessment relative to surgery, which could influence the results. Finally, the generalizability of our findings to other populations or surgical centers requires external validation. In addition, our definition of exercise intolerance relied solely on peak VO_2_. Other CPET parameters (e.g., anaerobic threshold, VE/VCO_2_ slope) were not included, as peak VO_2_ is the most established prognostic marker in Fontan patients. Future studies should incorporate a broader panel of CPET variables.

### 4.6. Clinical Implications and Future Directions

Despite these limitations, our findings carry significant clinical implications. They strongly advocate for “timely surgical management”, aiming to complete the TCPC before the age of 3.8 years where anatomically and clinically feasible, to optimize long-term functional outcomes. Furthermore, they highlight the critical importance of “meticulous perioperative and postoperative care” strategies aimed at minimizing complications and reducing ICU length of stay. This includes aggressive management of effusions, early extubation and mobilization, and optimal management of cardiac output.

Future research should be directed towards prospective, multi-center studies that incorporate longitudinal CPET assessments from a young age. These studies should integrate a broader array of variables, including advanced echocardiographic measures of systolic and diastolic function, magnetic resonance imaging for ventricular fibrosis and hemodynamics, and detailed assessments of physical activity, muscle mass, and psychosocial health. Such a holistic approach is essential to fully unravel the complex determinants of exercise intolerance and to develop targeted interventions to improve the quality of life for Fontan survivors.

## Figures and Tables

**Table 1:** Demographic Characteristics of Patients with Normal Exercise Tolerance Group vs. Decreased Exercise Tolerance Group.

Influencing Factor	ET Group (*n* = 45)	EI Group (*n* = 54)	*t*	*p*
Age at surgery (years)	3.60 (2.97, 4.59)	4.67 (3.36, 6.68)	−2.96	0.004 *
Male [*n* (%)]	33 (73.3)	33 (61.1)	1.65	0.20
Female [*n* (%)]	12 (26.7)	21 (38.9)	1.63	0.20
Preoperative height (cm)	95 (90, 100)	100 (93.75, 111)	−2.91	0.01 *
Preoperative weight (kg)	13.5 (12, 15.15)	14.55 (13.28, 18.9)	−2.55	0.01 *
Preoperative BMI (kg/m^2^)	15 (14.1, 16.15)	14.8 (14.08, 15.65)	−0.84	0.41
Preoperative BSA (m^2^)	0.63 (0.6, 0.77)	0.58 (0.54, 0.65)	−3.18	0.02 *
BMI at CPET (kg/m^2^)	14.8 (14.17, 16.11)	16.03 (14.06, 17.7)	−1.89	0.06
BSA at CPET (m^2^)	0.93 (0.82, 1.09)	1.04 (0.81, 1.34)	−1.52	0.127
Age at CPET (years)	8.65 (7.01, 13.81)	10.11 (7.68, 13.81)	−2.85	0.01 *
Follow-up time (years)	5.02 ± 2.25	5.32 ± 2.65	−2.51	0.66
Follow-up age [*n* (%)]				
≥10 years	11 (24.4)	28 (51.9)	7.72	0.005 *
<10 years	34 (75.6)	26 (54.5)		
NYHA Class, *n* (%)				
Class I	36 (80%)	36 (66.7%)		
Class II	9 (20%)	18 (33.3%)		

Note: *p* < 0.05 indicates statistical significance. Follow-up age is defined as the time interval between the last CPET and the TCPC surgery; Age at CPET refers to the age at the last CPET test; BSA: Body Surface Area; BMI: Body Mass Index; CPET: Cardiopulmonary Exercise Testing. NYHA: New York Heart Association. Follow-up time is presented as ($\bar{x}$ ± s); gender is expressed as n (%), and other data are presented as P50 (P25, P75). “*” was considered statistically significant.

**Table 2:** Perioperative Conditions of Children with Normal vs. Decreased Exercise Tolerance After Single Ventricle Fontan Surgery.

Influencing Factor	ET Group (*n* = 45)	EI Group (*n* = 54)	Statistic	*p*
Preoperative oxygen $\mathrm{saturation}\;[\%,\;\bar{x}$ ± s]	79.40 ± 8.09	78.08 ± 7.36	−0.51	0.62
Last NT-proBNP	136.4 (92.22, 280.7)	113.7 (62.99, 422.13)	−0.50	0.62
Complicated with pulmonary hypertension [*n* (%)]	3 (6.7)	5 (9.2)	0.19	0.66
Tricuspid atresia [*n* (%)]	7 (15.6)	11 (20.4)	0.38	0.54
Complicated with visceral heterotaxy syndrome [*n* (%)]	8 (17.8)	13 (24.1)	0.58	0.45
Pulmonary artery condition [*n* (%)]				
Pulmonary artery or valve stenosis	22 (48.9)	34 (63)	2.08	0.35
Pulmonary artery atresia	16 (35.6)	13 (24.1)		
Normal pulmonary artery	7 (15.6)	7 (13)		
Atrioventricular valve regurgitation [*n* (%)]				
Mild regurgitation	18 (40)	22 (41.5)	2.87	0.41
Moderate regurgitation	3 (6.7)	8 (15.1)		
Severe regurgitation	1 (2.2)	0 (0)		
No regurgitation	23 (51.1)	23 (43.4)		
Intraoperative fenestration [*n* (%)]	40 (88.9)	48 (88.9)	<0.01	1.00
Operation duration [min, $\bar{x}$ ± s]	267.53 ± 93.03	312.14 ± 89.79	−2.554	0.01 *
Cardiopulmonary bypass time [min, $\bar{x}$ ± s]	103.59 ± 48.46	122.26 ± 58.49	−2.006	0.045 *
Postoperative ventilator time ≥ 48 h [*n* (%)]	2 (4.4)	2 (3.7)	0.035	0.85
ICU stay > 3 days [*n* (%)]	14 (31.1)	31 (68.9)	6.85	0.01 *
Postoperative hospital stay >14 days [*n* (%)]	20 (44.4)	25 (55.6)	3.40	0.06
Chest tube duration > 2 weeks or chylothorax [*n* (%)]	15 (40.5)	22 (59.5)	0.58	0.45
Postoperative reoperation [*n* (%)]	3 (25)	9 (75)	2.30	0.30
Unplanned postoperative hospitalization [*n* (%)]	8 (32)	17 (68)	2.44	0.12
Postoperative ECG abnormalities [n (%)]	20 (37.7)	33 (62.3)	3.52	0.06
Moderate or severe AV valve regurgitation [*n* (%)]	6 (14.3)	16 (33.3)	4.40	0.04 *
Postoperative complications [*n* (%)]	14 (34.1)	27 (65.9)	3.61	0.06

Note: *p* < 0.05 indicates statistical significance. Operation duration, cardiopulmonary bypass time, and preoperative oxygen saturation are presented as ($\bar{x}$ ± s); other data are presented as P50 (P25, P75). “*” was considered statistically significant.

**Table 3:** Area under the receiver operating characteristic curve (AUC) for predictive variables.

Variable	AUC	Standard Error	*p* Value	95% Confidence Interval
Surgical Age	0.692	0.053	0.001 *	0.588–0.795
Examination Age	0.644	0.055	0.014 *	0.536–0.753

Note: *p* < 0.05 was considered statistically significant. “*” was considered statistically significant.

**Table 4:** Univariate and Multivariate Logistic Regression Analyses of Factors Associated with Decreased Exercise Tolerance after TCPC Surgery.

Variable	Univariate Analysis		Multivariate Analysis	
	OR (95% CI)	*p*	aOR (95%CI)	*p*
Age at TCPC surgery ≥ 3.8 years	3.95 (1.71–9.72)	**0.001**	2.54 (1.01–6.42)	**0.049**
Age at last CPET ≥ 10 years	3.33 (1.40–7.90)	**0.006**	1.89 (0.70–5.13)	0.21
Operation duration ≥ 237.5 min	5.35 (2.20–13.00)	**<0.001**	2.93 (0.95–8.98)	0.06
Cardiopulmonary bypass time ≥ 77.5 min	3.33 (1.31–8.46)	**0.01**	1.59 (0.47–5.35)	0.45
ICU stay > 3 days	2.98 (1.30–6.85)	**0.01**	2.61 (1.07–6.39)	**0.04**
Postoperative moderate/severe AV regurgitation	4.30 (0.88–21.05)	**0.048**	2.38 (0.45–12.77)	0.31
Postoperative ECG abnormalities	2.17 (0.96–4.91)	0.06	-	-
Any postoperative complications	2.21 (0.97–5.06)	0.06	-	-
Postoperative hospital stay > 14 days	2.13 (0.95–4.76)	0.06	-	-

Abbreviations: AV, atrioventricular; CPET, cardiopulmonary exercise test; ICU, intensive care unit; OR, odds ratio; aOR, adjusted odds ratio; CI, confidence interval. Note: The outcome variable is decreased exercise tolerance. All variables listed in the univariate analysis were considered for the multivariate model. The final multivariate logistic regression model included variables with a *p* value < 0.05 from the univariate analysis (as indicated). A dash (-) indicates that the variable was not included in the final multivariate model. Bolded *p* values indicate statistical significance (*p* < 0.05). Multivariate model C-statistic = 0.765 (95% CI 0.68–0.85); Hosmer–Lemeshow χ^2^ = 6.84, *p* = 0.41, indicating good discrimination and calibration.

## Data Availability

The de-identified participant data that underpin the results reported in this article will be made available upon reasonable request to the corresponding author, subject to the approval of the institutional ethics committee and the execution of a data sharing agreement.

## Abbreviations

The following abbreviations are used in this manuscript:

TCPC	Total cavopulmonary connection
CPET	Cardiopulmonary exercise testing
BMI	Body mass index
BSA	Body surface area
